# Effect of Morphology and Plasmonic on Au/ZnO Films for Efficient Photoelectrochemical Water Splitting

**DOI:** 10.3390/nano11092338

**Published:** 2021-09-08

**Authors:** Mohamed Zayed, Nourhan Nasser, Mohamed Shaban, Hind Alshaikh, Hany Hamdy, Ashour M. Ahmed

**Affiliations:** 1 Nanophotonics and Applications (NPA) Laboratory, Physics Department, Faculty of Science, Beni-Suef University, Beni-Suef 62514, Egypt; m.zayed88ph@yahoo.com (M.Z.); Nnourhan673@gmail.com (N.N.); hshamdy@hotmail.com (H.H.); ashour.elshemey@gmail.com (A.M.A.); 2 Department of Physics, Faculty of Science, Islamic University in Madinah, Al-Madinah Al-Munawarah 42351, Saudi Arabia; 3 Chemistry Department, Science and Arts College, Rabigh Campus, King Abdulaziz University, Jeddah 21911, Saudi Arabia; hfalshakh@kau.edu.sa

**Keywords:** ZnO nanostructures, Au Surface plasmonic, photoelectrochemical, water splitting

## Abstract

To improve photoelectrochemical (PEC) water splitting, various ZnO nanostructures (nanorods (NRs), nanodiscs (NDs), NRs/NDs, and ZnO NRs decorated with gold nanoparticles) have been manufactured. The pure ZnO nanostructures have been synthesized using the successive ionic-layer adsorption and reaction (SILAR) combined with the chemical bath deposition (CBD) process at various deposition times. The structural, chemical composition, nanomorphological, and optical characteristics have been examined by various techniques. The SEM analysis shows that by varying the deposition time of CBD from 2 to 12 h, the morphology of ZnO nanostructures changed from NRs to NDs. All samples exhibit hexagonal phase wurtzite ZnO with polycrystalline nature and preferred orientation alongside (002). The crystallite size along (002) decreased from approximately 79 to 77 nm as deposition time increased from 2 to 12 h. The bandgap of ZnO NRs was tuned from 3.19 to 2.07 eV after optimizing the DC sputtering time of gold to 4 min. Via regulated time-dependent ZnO growth and Au sputtering time, the PEC performance of the nanostructures was optimized. Among the studied ZnO nanostructures, the highest photocurrent density (J_ph_) was obtained for the 2 h ZnO NRs. As compared with ZnO NRs, the J_ph_ (7.7 mA/cm^2^) of 4 min Au/ZnO NRs is around 50 times greater. The maximum values of both IPCE and ABPE are 14.2% and 2.05% at 490 nm, which is closed to surface plasmon absorption for Au NPs. There are several essential approaches to improve PEC efficiency by including Au NPs into ZnO NRs, including increasing visible light absorption and minority carrier absorption, boosting photochemical stability, and accelerating electron transport from ZnO NRs to electrolyte carriers.

## 1. Introduction

Energy is one of the most pressing issues confronting humanity in the 21st century. Most of world’s energy (approximately 80%) originates from the burning of fossil fuels such as oil, coal, and natural gas. Unfortunately, these fossil fuels have many drawbacks [1]. Fossil fuels are non-renewable sources fuels, and within a limited period, they will inevitably run out. The combustion of fossil fuels is followed by CO_2_ emissions due to the reaction between carbon in fossil fuels and O_2_ gas. This leads to a reduced amount of oxygen in the atmosphere and threatening life on earth [2]. The CO_2_ gas emitted into the atmosphere often increases temperatures and triggers greenhouse effects and climate change [3]. It is, therefore, very important to use all the available renewable energy sources to address these problems [4,5].

On the other side, hydrogen gas is a versatile energy carrier. As H_2_ is burned with O_2_, H_2_O is created by releasing energy. In short, as an alternative energy source to substitute fossil fuels, hydrogen-based fuels have gained much attention. Hydrogen fuel is sustainable, clean, produces carbon-free emissions, and has high efficiency for energy conversion [3]. It can also help to address several major energy challenges. On earth, hydrogen is an accessible element, but it is not present as a free molecule. There are many methods for hydrogen production, such as methane reforming, biomass gasification, thermal cracking, electrolysis, photolysis, pyrolysis, and the photoelectrochemical method. Among them, the photoelectrochemical (PEC) method is a low cost, eco-friendly, high performance, and less energy-intensive process [6,7,8,9]. The light is absorbed by the photocatalyst via this technique and used to induce chemical reactions including water splitting to generate hydrogen and oxygen. There are several semiconductor metal oxides, such as NiO [10], ZnO [11], Cu_2_O [12], TiO_2_ [13], SnO_2_ [13], WO_3_ [14], SrTiO_3_ [15], Fe_2_O_3_ [16], BiVO_4_ [17], and Ta_2_O_5_ [18], that have been improved and applied as photocatalysts for PEC water splitting. Among these metal oxides, ZnO (n-type) semiconductor with a direct bandgap of nearly 3.36 eV [19,20,21,22]. Additionally, ZnO has excellent chemical stability [23,24,25], good electron mobility [26], very high exciton binding energy, and good compatibility with the environment [27]. Zinc oxide is a multifunctional material, which means that it has many areas of applications, such as optoelectronics, electronics, adsorption, sensors, photocatalysis, and energy conversion [28,29]. Additionally, it has many biomedical applications, for instance in microbial killing, drug delivery, photodynamic therapy, wound healing, and disease diagnosis [28,29,30]. Hence, ZnO nanomaterials with many morphologies have emerged as promising PEC photoelectrodes.

There are several methods to fabricate ZnO nanostructures, such as hydrothermal, thermal evaporation, electrochemical, sol-gel, atomic layer deposition, chemical vapor deposition, pulsed laser ablation, and a microwave synthesis method [28,31,32,33,34,35,36,37]. These methods can be used to fabricate ZnO of different nanomorphologies, such as nanorods, nanodiscs, nanotrees, nanosheets, nanoflowers, nanotriangles, nanowires, nanopencils, and nanotubes [38,39,40,41,42,43]. The methods used to design controlled nanomorphologies, however, are complicated processes, time-consuming, and require complicated devices. Hence, it is essential to utilize a simple, effective, and inexpensive preparation route for ZnO of controlled nanomorphologies.

Research studies based on ZnO nanostructures for enhancement of PEC water splitting have recently been carried out. Rekha et al. reported that the J_ph_ was approximately 0.39 mA/cm^2^ for ZnO NPs [44]. Basu et al. used Bi_2_S_3_/ZnO heterojunctions to improve PEC performance [45]. Hsu et al. prepared Fe_2_O_3_/ZnO core–shell to increase the efficiency of PEC water splitting [46]. Kim et al. reported ZnS/ZnO to essentially prevent photo-corrosion [47]. Kang et al. showed that the deposition of Au NPs on ZnO NWs exhibited a J_ph_ of 1.6 mA/cm^2^ [48]. However, the reported J_ph_ values of these studies are very small, and the conversion efficiencies are very limited. The photocorrosion and short lifetime of free charge carriers of the ZnO nanostructures continue to limit the stability and efficiency of the PEC water splitting [49,50,51,52,53,54].

The coupling of plasmonic NPs (especially Au) with a semiconductor surface is a good approach to enhance photoelectric conversion for many reasons as follows [55,56,57,58,59]. The formation of semiconductor–metal junction helps in minimizes electron–hole recombination. It can enhance the absorption of light in the visible domain according to surface plasmon resonance (SPR) of the gold (Au) NPs. The SPR increases the charge carriers’ density and facilitates their transportation. Additionally, Au NPs protect the coated ZnO layer from photocorrosion during the PEC reaction [60]. The electronic properties at the interfaces between ZnO and Au were modified due to an electromagnetic plasmon–exciton coupling [61]. This work aims to design ZnO PEC electrodes with controlled nanomorphologies (NRs, NRs/NDs, and NDs) and Au/ZnO with Au of different thicknesses to enhance their PEC performance. Different performance indicators have been studied, such as J_ph_ values, conversion efficiencies, and number of hydrogen moles.

## 2. Experimental Details

The growth in the ZnO nanostructures was carried out by combining the successive ionic-layer adsorption and reaction (SILAR) with the chemical bath deposition (CBD) method. The advantages of these methods are simple, rapid, low-cost, and do not require any complex instruments [62,63]. The ZnO nanostructure was decorated with Au NPs of different deposition times using the direct current (DC) sputtering technique.

### 2.1. Materials

Zinc acetate (Zn(CH_3_CO_2_)_2_) (99%, Alfa Aesar, Fisher Scientific, ON, Canada), acetone (C_3_H_6_O) (Alfa Aesar), Zinc sulfate (Zn(SO_4_)) (99%, Sigma-Aldrich Inc., Darmstadt, Germany), methanol (CH_4_O) (98%, Alfa Aesar), sodium hydroxide (NaOH) (99.6%, Sigma-Aldrich Inc.), ammonia (NH_4_OH) (30%, Sigma-Aldrich Inc.), hexamethylene tetra ammine (HMT) (99.98%, Alfa Aesar), and hydrochloric acid (HCl) (98%, Sigma-Aldrich Inc.) were used to prepare the films.

### 2.2. Fabrication of Pre-Layer ZnO

A commercial glass substrate with dimensions 1.0 × 2.0 × 0.11 cm^3^ was cleaned in boiling sulfuric acid (1:15 *v*/*v*) for 30 min and then rinsed several times in acetone, ethanol, and distilled water (DW). The SILAR method is composed of many parts, including the immersion of a substrate (glass) vertically in cationic and anionic solutions. According to the SILAR method, 120 mL of 0.05 M of Zn(SO_4_) with 30 mL ammonia (30%) solution (cationic source) and hot DW at 70 °C (anionic source) were used. Firstly, the glass substrate was immersed for 40 s into a cationic precursor, so zinc ammonia complex ([Zn (NH_3_)_4_]^2+^) was adsorbed onto the glass substrate. Secondly, the complex zinc ammonia substrate was immersed into a DW for 20 s at room temperature, wherever the zinc ammonia layer transformed into zinc hydroxide (Zn (OH)_2_). Thirdly, the coated substrate with Zn(OH)_2_ was exposed to ultrasonic (ultrasonic cleaner with power 100 W, MTI corporation, model UD100SH-3.8Q, USA) for 50 s to remove the weakly bonded zinc hydroxide molecules. Finally, the Zn(OH)_2_ substrate was rinsed into the DW and kept at 70 °C for 20 s, where zinc hydroxide Zn (OH)_2_ converted into ZnO. The chemical reaction formula for ZnO nanostructures growth is described simply by Reactions (1)–(4):

(1) $${\mathrm{ZnSO}}_{4}+4{\mathrm{NH}}_{3}H_{2}O\to \;{\left[\mathrm{Zn}({\mathrm{NH}}_{3}{)}_{4}\right]}^{2+}+H_{2}{\mathrm{SO}}_{4}$$

(2) $${\left[\mathrm{Zn}({\mathrm{NH}}_{3}{)}_{4}\right]}^{2+}+4H_{2}O\;\to {\mathrm{Zn}}^{2+}+4{\mathrm{NH}}_{4}+4{\mathrm{OH}}^{-}$$

(3) $${\mathrm{Zn}}^{2+}+2{\mathrm{OH}}^{-}\;\to \mathrm{Zn}(\mathrm{OH}{)}_{2}$$

(4) $$\mathrm{Zn}(\mathrm{OH}{)}_{2}\;\to \mathrm{ZnO}+H_{2}O$$

Thus, only one cycle for ZnO deposition was achieved. A drying period of 30 s at 80 °C was sustained before the start of another SILAR cycle. The fabricated ZnO seed layer was heated in a muffle furnace (Thermo Scientific Thermolyne, model F6010, Walthman, MA, USA) for 1 h at 300 °C in atmospheric air. The optimum conditions here for the seed layer were set at 20 cycles.

### 2.3. Synthesis of Hierarchical ZnO Thin Film

The hierarchical ZnO nanostructured thin films were deposited over the seed layer by the CBD method. Equi-molar (0.05 M) of Zinc acetate and HMT were dissolved in 50 mL of DW under stirring. The pH of the bath reaction was set to be 6 by adding 0.1 M NaOH, dropwise. The glass, covered by a ZnO seed layer, was dipped vertically in a chemical bath solution at 80 °C. The time of deposition was controlled at 2, 6, and 12 h to fabricate ZnO nanostructures with different morphology. The fabricated ZnO films were cleaned with DW and acetone several times to remove any remaining salts. Finally, the films were dried for further characterization and application.

### 2.4. Decoration of ZnO Films with Au NPs

From the optical properties and data of PEC in the following sections, the ZnO surface NRs at a deposition time of 2 h is considered as the best-optimized behavior. Hence, ultrathin Au layers were sputtered on this film using DC sputtering (LA440S Ardenne). The time of sputtering was changed (1 to 4 min) to get several Au thicknesses. Au layers were sputtered using a high purity gold target (Au, 99.999%) onto ZnO/glass films. Au deposition was performed at the pressure 2 Torr and current 0.015 A. Under the same conditions, the cleaned glass substrate was coated with Au layers directly to realize the influence of Au SPR on the properties of ZnO films. The rate of growth of the Au layer was 5 nm/min.

### 2.5. Characterization Techniques

X-ray diffraction (XRD, Bruker/Siemens D5000) with high-resolution was used for identifying the structure properties for ZnO samples using Cu Kα radiation (λ = 1.5418 Å). The chemical compositional information was examined using energy dispersive X-ray spectroscopy analysis (EDX, INCA x-act, Oxford Instruments, Concord, MA, USA). Morphological studies of the ZnO thin films were investigated by using field emission scanning electron microscopy (FE-SEM, Zeiss GeminiSEM 360, ZEISS Microscopy, Munich, Germany). A spectrophotometer (LAMBDA 950 UV/Vis/NIR, PerkinElmer Inc., Waltham, MA, USA) was used to examine the optical properties.

### 2.6. Photoelectrochemical (PEC) Measurements

The PEC analysis was examined by using a Keithley 2400 Sourc-emeter (Tektronix, Beaverton, OR, USA) in the two-electrodes system. The prepared ZnO and Au/ZnO films acted as the working electrode. The silver paste was used to a created ohmic electrical contact on the surface of the ZnO and Au/ZnO electrodes. A platinum foil served as a counter electrode. The photocurrent was recorded under irradiation by a 400 Watt Xenon lamp (Newport Corporation, Newport, UK) as the simulative solar light.

The voltage (V) was scanned from −1 to + 1 V. An aqueous solution of 0.3 M Na_2_S_2_O_3_ was used as a supporting electrolyte medium (pH = 7.0) that consumes the photo-generated holes from the photocatalytic surface.

## 3. Results and Discussion

### 3.1. Characterization of the Fabricated ZnO Nanostructured Thin Film

#### 3.1.1. Structural Properties

**Figure 1:**
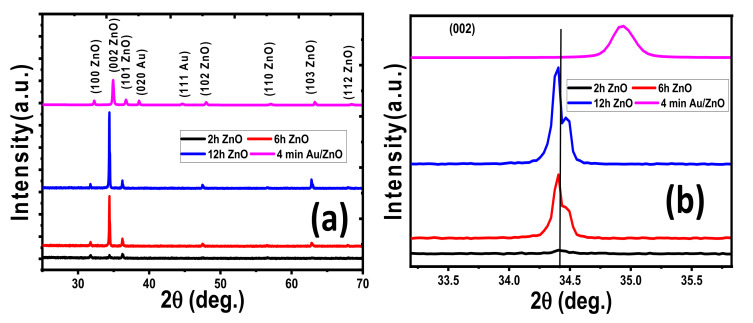
(**a**) XRD patterns for pure nanostructured ZnO films prepared at different deposition times and 4 min Au/ZnO film, and (**b**) the peak position for the plane (002).

XRD analysis has been performed for fabricated ZnO nanostructure samples to identify the crystal structure and chemical composition. Figure 1 shows the XRD charts of ZnO nanostructures deposited at different time deposition of 2, 6, and 12 h on a glass substrate. Based on standard cards JCPDS-89–0510, all fabricated samples show polycrystalline structures with the diffraction patterns of the hexagonal phase of wurtzite ZnO (space group C6V = P63mc) [64,65]. Additionally, all prepared films display a sharp diffraction peak (002) at 2θ = 34.4° and grow preferably perpendicular to the surface of the glass through the c-axis. The other minor peaks are located at 2θ = 31.72° and 36.21°, 47.45°, 56.58°, 62.89°, and 67.86°, attributed to Miller’s indexes (100), (101), (102), (110), (103), and (112) planes of ZnO, respectively. This means that the deposition time did not affect the ZnO film hexagonal structure. In addition, the XRD pattern does not detect any additional impurity phases for Zn metal or Zn compounds. This implies the creation of pure crystalline ZnO thin films by CBD. Even though ZnO was fabricated at low temperatures, the sharp and strong diffraction peaks indicate good crystallization of the samples. With increases in deposition time to 2 and 12 h, the (002) peaks became sharper and stronger, which suggests the better growth and crystallization of the prepared ZnO nanostructured films. This may be attributed to the thickness of films rising as the deposition time increases. In several previous studies, the (200) was observed as preferential growth for ZnO films [66]. The (002) peak has higher intensities for all ZnO films, which suggests a preferential orientation due to the c-axis. This is correlated with the smallest surface free energy value for the (002) plane compared to other planes, increasing the number of grains along this plane [67]. For (002), (110), and (100) planes of ZnO, the surface free energy is 9.91, 12.32, and 20.91 eV/nm^2^, respectively [68].

From Figure 1b, the (002) peak location is shifted to lower 2θ values with increasing deposition time due to induced structural lattice deformation. Hence, the inter-planar distance for (002) planes was increased as displayed in Table 1 because the diffraction angle is inversely related to the inter-planar distance due to Bragg’s equation

(5) m λ=2 d sinθ

where λ is the incident X-ray wavelength, d is the inter-planar distance, and m is the order of diffraction. θ is Bragg’s diffraction angle. The crystallite size (D) of the ZnO films was calculated from the full width at half maximum intensity (β) using the Debye–Scherrer formula, D = 0.9 λ ÷ β cosθ, where λ = 0.154 nm. The crystallite size decreased from approximately 79 to 77 nm as time increased from 2 to 12 h for the main peak (002), as listed in Table 1. This is due to the fact that the crystallites smaller in size may tend to accumulate with increasing the time of deposition.

The lattice constants of the ZnO films (a and c), the unit cell volume (V) of the hexagonal ZnO, internal parameter (u), Zn-O bond length (L), and the stress (σ) of the fabricated samples are calculated by the next Equations [69,70,71,72];

(6) $$d_{\left(h\;k\;l\right)}={\left[\frac{4}{3}\left(\frac{h^{2}+k^{2}+h\;k}{a^{2}}\right)+\frac{l^{2}}{c^{2}}\right]}^{-0.5}$$

(7) $$V=\frac{\sqrt{3}}{2}{\;a}^{2}\;c$$

(8) $$u=\left(\frac{1}{3}\right)\left(\frac{a^{2}}{c^{2}}\right)+\frac{1}{4}$$

(9) $$L=\sqrt{\left(\frac{a^{2}}{3}+(\frac{1}{2}-u{)}^{2}c^{2}\right)}$$

(10) $$ℴ=4.5\;\times \;10^{11}\;\frac{c_{o}-c}{c_{o}}\;\;N/m^{2}$$

The effect of time could have caused internal stress in ZnO films and, in turn, the blue shift in the diffraction peak occurred. Hence, the lattice parameters and the unit cell volume of ZnO films are affected as a result of a change in strain levels in both samples [71]. From Table 1, the parameter is increased with deposition time. The V value is 47.5176 Å^3^ for the ZnO film deposited at 2 h. Then, as the time of deposition increased from 6 to 12 h, the V value of the ZnO samples decreased from 47.7392 to 47.6748 Å^3^. The Zn-O bond length exhibits a similar behavior to that seen in Table 1. The L value of deposited ZnO increased from 1.975 to 1.978 Å by increasing the time of deposition from 2 to 6 h, and then it decreased to 1.976 Å for the film deposited at 12 h. The stress (σ) value changed from −2.0910 × 10^8^ to −5.17566 × 10^8^ N/m^2^ as the time deposition increased from 2 to 6 h. The behavior of σ is dependent on the value of the lattice constant (c) of the ZnO film. The negative sign of the estimated stress values shows that the ZnO crystallites are under compressive stress (tensile) in all samples.

**Figure 2:**
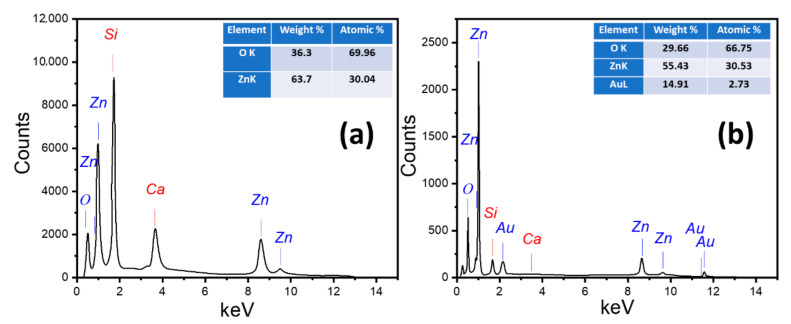
EDX patterns for nanostructured (**a**) pure ZnO and (**b**) Au/ZnO thin film.

The phase structure of the ZnO crystal was not considerably changed by the Au deposition, as shown in Figure 1. Two additional small peaks appeared at 2θ = 38.56° and 44.63° for the 4 min Au/ZnO NRs film, attributed to Miller’s indexes (020) and (111), respectively. These peaks are for the cubic Au as indexed to its crystalline data (JCPDS card no. 96–901–1613, a = b = c = 4.065 Å). After 4 min Au coating, the position of the main peak (002) shifts to higher 2θ values due to induced structural lattice deformation, as seen in Figure 2b. The primary peak’s intensity (002) is reduced, while the full width at half maximum intensity (β) is increased. From Table 1, the crystallite size for the main peak (002) decreases from approximately 79 nm for ZnO NRs to 65 nm for 4 min Au/ZnO NRs due to the increase in β value. As shown in Table 1, the decrease in d-spacing for 4 min Au/ZnO NRs (2.57 Å) compared to pure ZnO NRs (5.2976 Å) is due to the peak shifting towards a higher Bragg angle.

#### 3.1.2. Chemical Composition

The energy dispersive X-ray (EDX) method is commonly employed for quantitative elemental information. It is considered a low-cost, rapid, and non-destructive technique for surface analysis. Figure 2 displays the EDX spectra for the ZnO nanostructured film deposited at 2 h and 4 min Au/ZnO film. The inset tables give the quantitative analysis of the chemical composition of the pure ZnO and Au/ZnO thin films. Figure 2a shows the EDX chart of pure ZnO, which approves the presence of Zn and O peaks. Only one peak for O is located at 0.75 keV. There are four peaks related to Zn at 0.91, 1.0, 8.5, and 9.6 keV. The quantitative analysis for ZnO film is 63.7 and 36.3% for Zn and O, respectively. This shows the high purity of the prepared ZnO films, which matches with XRD data. The obtained Zn and O atomic ratios depart from the ZnO stoichiometric ratio. It is crucial to notice that the oxygen signal is not only received from the ZnO nanostructure, but also from the surface of the glass substrate, as indicated in Figure 2a by the presence of a signal for Si and Ca. This indicated that the thickness of ZnO nanostructured film is smaller than the EDX interaction volume (≥1 cube µm^3^).

Figure 2b shows the EDX pattern for 4 min Au/ZnO NRs, with an inset table showing the chemical composition quantitatively. The existence of Zn, Au, and O peaks is confirmed by the EDX spectrum. The weight % for Zn, O, and Au signals of the 4 min Au/ZnO NRs film were found to be 55.43, 29.66, and 14.91%, respectively.

#### 3.1.3. Morphological Analysis

**Figure 3:**
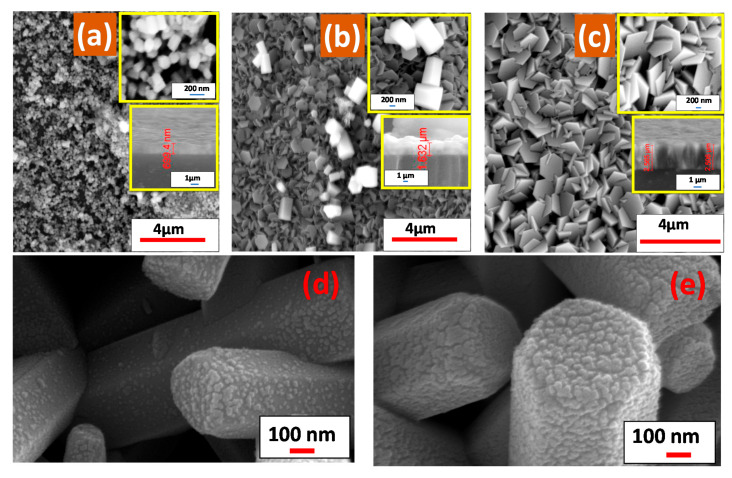
SEM images for ZnO films fabricated at different growth times: (**a**) 2, (**b**) 6, and (**c**) 12 h. SEM images for Au/ZnO films with different Au sputtering time: (**d**) 2 and (**e**) 4 min.

Figure 3 displays SEM images of ZnO films deposited on the ZnO seed layer by the CBD method at different reaction times. The morphology of ZnO nanostructures undergoes a gradual evolution with deposition time, as presented in Figure 3. Random distribution of ZnO NRs was grown at a deposition time of 2 h (Figure 3a). The average length and diameter of these NRs are 611 and 210 nm, respectively. The random growth of NRs is dependent on the polycrystalline glass effect. All samples of ZnO NRs have a hexagonal structure with a regular shape and grow preferentially in the c-axis direction as obtained from XRD analysis. Figure 3b shows the SEM image of the ZnO film deposited at 6 h. The morphology of the ZnO film contains both a huge number of hexagonal nanodisc and a very small number of hexagonal NRs. The ZnO NDs have random distributions and directions. The average values of the hexagonal side length and diameter of ZnO NDs are approximately 360 and 380 nm, respectively. At the deposition time of 12 h, the NRs had disappeared, and the ZnO nanostructures had completely become hexagonal nanodiscs, as shown in Figure 3c. The NDs interfered with each other, and some cracks appeared with a high cover density of glass substrate. The average values of the ZnO NDs side length and diameter are approximately 410 and 1100 nm, respectively. The obtained dimensions of ZnO nanostructures using SEM images are larger than the obtained XRD crystallite size, indicating that the nanostructures are composed of more than two crystallites.

Figure 3d,e shows the morphology of Au/ZnO films with various Au deposition times (2 and 4 min). Individual Au nanoparticles are deposited with random distribution on the surface of the ZnO nanorods for 2 min deposition, as shown in Figure 3d. The size of the Au nanoparticles ranges from 7 to 20 nm. More Au nanoparticles are deposited when the deposition time is increased to 4 min, Figure 3e. As a result, the nucleation and growth of Au nanoparticles are increased, resulting in Au nanograins with a rough surface. The Au nanograins have nearly completely coated the surfaces of ZnO NRs. The average size of Au nanograins is approximately 26 ± 4 nm.

Figure 3 shows the variance in the thickness of the film with time deposition. The sample thickness increased from 0.700 µm at 2 h to 1.642 µm at 6 h and 2.496 µm at 12 h.

SEM images in Figure 3a–c reveal that ZnO nanostructures films changed from hexagonal NRs into hexagonal NDs as the time deposition increased from 2 to 12 h. The preparation of ZnO nanostructures with control of their shape has been one of the main objectives in novel applications. In the previous analysis, the same result was achieved by increasing the concentration of OH^−^ in the precursor [73]. At low OH^−^ concentrations in the precursor solution, the growth rate of ZnO crystal is faster along the c-axis compared to other any orientation. Therefore, the ZnO thin films exhibit one-dimensional (1D) NR morphology with (002) favored orientation, according to the high surface energy. By increasing the OH^−^ concentration, more OH^−^ ions ligands to the (002) plane of ZnO partially prevent the ZnO crystal growth along the c axis [69]. Therefore, the ZnO crystallites can still expand sideways, and hence, two-dimensional (2D) ZnO NDs are produced. Ammonia (NH_3_) reacts with water (H_2_O) during the growth process to produce ammonium hydroxide (NH_4_OH), which is considered a continuous hydroxide ion (OH^−^) source for hydrolysis. As the growth time of ZnO films increased, the OH^−^ concentration in the solution increased. Therefore, the ratio (height/width) for ZnO NRs is decreased. Hence, the ZnO nanostructures are converted from one-dimensional hexagonal NRs into two-dimensional hexagonal NDs.

#### 3.1.4. Optical Properties

##### Effect of Time Deposition

**Figure 4:**
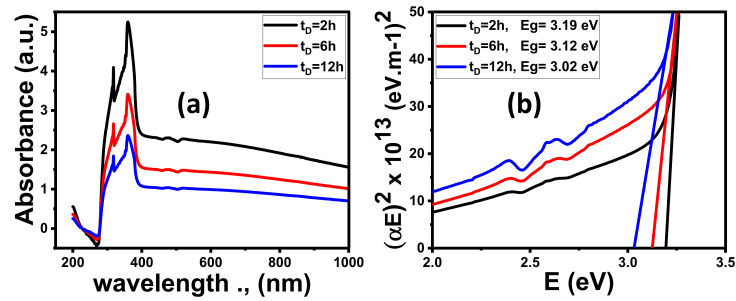
Optical spectra of ZnO thin films fabricated at different times; (**a**) absorbance and (**b**) (αhν)^2^ versus hν.

Optical spectroscopy is a powerful method to examine the optical properties and energy bandgap of nanomaterials in the UV–visible region. The absorbance (A%) pattern of ZnO films deposited at different times on the substrates is demonstrated in Figure 4a. The ZnO films show strong absorption bands in the UV region resultant in electron jump from the valence band to the conduction band of ZnO (O^2p^ → Zn^3d^). The absorbance decreases sharply from the ultraviolet (UV) to the visible region (vis). The absorbance values are significantly affected by ZnO film morphology. The NR morphology deposited at 2 h showed the highest absorbance. As the NDs are deposited at 12 h, the lowest absorbance is obtained. For wavelengths above 400 nm, the absorbance values of all films are nearly constant. As the deposition time increased from 2 to 12 h, the absorbance values decreased from 18 to 40%. The right side of the UV absorption band is moved toward lower wavelengths. This implies that the band gap (Eg) for ZnO films shifted to the visible region.

The absorption coefficient (α) was computed by using the measured absorbance (A) data, and film thickness (d) from the following equation:

(11) $$\alpha =\left(2.303/d\right)\;A\;$$

For the direct transition of electrons from the VB to CB, the optical bandgap energy can be expected using the Tauc equation:

(12) $${\left({\alpha \;E}_{\mathrm{ph}}\right)}^{2}=\;B\;{\left(E_{\mathrm{ph}}-{\;E}_{g}\right)}^{\;}$$

where Eph = hυ is the energy of an incident photon. Eg is the calculated bandgap energy and B is the independent energy constant.

The energy intercept of the plots of (α Eph)^2^ versus (hν) can be given the Eg for a direct transition (Eg = Eph at α = 0). As shown in Figure 4b and Table 2, the Eg of ZnO film decreased from 3.19 to 3.02 eV as the deposition time increased from 2 to 12 h. This blue shift is due to a change in the ZnO nanostructured morphology from NRs to NDs. The optical features of the ZnO NRs films that are deposited at 2 h have the best optical properties compared to other samples.

##### Effect of Coating Au Thickness

The values of the Eg suggest that the three ZnO nanomorphologies are efficient UV absorbers and weak absorbers for light in the visible range, which agrees with absorbance, as shown in Figure 4a. This imposes a restriction on ZnO for water splitting efficiency. Therefore, the ZnO NRs samples that were fabricated at 2 h were enhanced by coating with Au nanoparticle (NPs) by controlling Au coating time (1, 2, 3, and 4 min) using DC sputtering.

**Figure 5:**
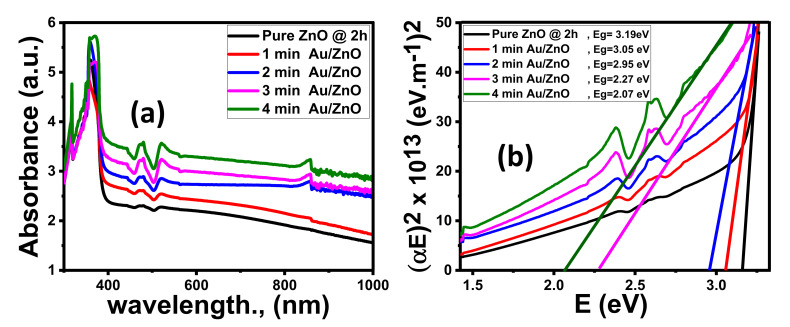
Optical spectra of Au/ZnO NRs; (**a**) absorbance and (**b**) (αhν)^2^ versus hν.

As the coating time increased, the thickness of the Au NPs increased and the color of pure ZnO films varied from white to gray. As clearly presented in Figure 5a, the absorbance of ZnO films greatly depends on the Au sputtering time (thickness). By increasing the Au coated time, the absorption intensity increased gradually in the visible light region. This reveals the interaction between ZnO and Au. At λ = 490 nm, the absorbance intensity increases from 2.18% for pure ZnO film to 3.19% for 4 min Au/ZnO film. This is a result of Au’s complex refractive index and its huge imaginary part. The metal absorption is because the free electrons are excited from the conduction band (CB) to a higher Fermi level (EF) because of the phenomenon of surface plasmonic resonance (SPR) according to Au nanostructures [70]. The same behavior was observed for Al/ZnO, Ag/ZnO, and Pt/ZnO [74,75,76].

Using the Tauc equation, the Eg values decrease from 3.19 to 2.07 eV as Au time coating increased from 0 to 4 min, as shown in Figure 5b. Therefore, the position of Eg for 4 min Au/ZnO thin film is located in the visible light region, which covers a huge part of the solar radiation spectrum.

### 3.2. Photo-Electrochemical (PEC) Studies

**Figure 6:**
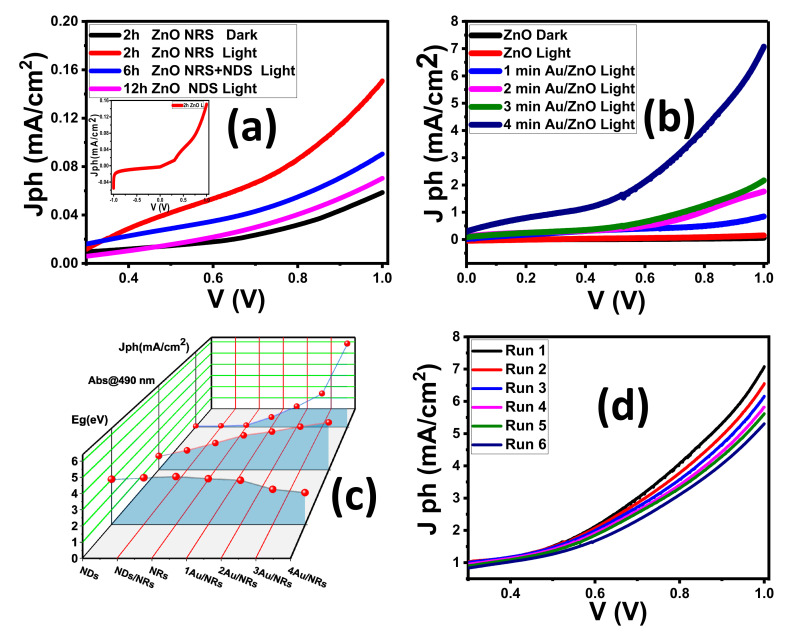
(**a**) PEC of ZnO thin films deposit at a different time (2, 6, 12 h); (**b**) PEC of Au/ ZnO NRs at different Au coted time (1, 2, 3, 4 min); (**c**) variation in the energy gap, absorbance at 490 nm, and photocurrent density at 1 V for all films; and (**d**) reproducible studies of J_ph_–V curves of Au/ZnO NRs.

The J_ph_–V characteristic curves show the highest J_ph_ values in the positive potential region as inset Figure 6a, indicating that the fabricated electrodes are n-type semiconductor characters with electrons as the majority free carriers.

Figure 6a shows the anodic photocurrent response curves of different ZnO nanomorphologies fabricated at different deposition times. Without illumination, the lowest value of a given dark current density (J_ph_) of all samples was nearly 40 µA/cm^2^ for pure ZnO (2 h), revealing that few chemical reactions occurred in dark. This suggests that there is an inadequate formation in the dark of photoinduced electron–hole pairs and a well-formed depletion layer, which is a typical feature of ZnO. Consequently, relative to the J_ph_ under illumination, the dark electrical current density is negligible. Low dark current density also refers to the ZnO electrodes; when submerged in the electrolyte, they are very stable.

The J_ph_ values observed under light illumination reveal that the incident photons increase the production of the charge carrier, which greatly contributes to the splitting of water.

The J_ph_ of the ZnO NRs deposited at 2 h (approximately 0.156 mA/cm^2^ at applied 1 V) is higher than other nanostructure ZnO. This means that the ZnO NRs had the highest level of generated photocurrent. This outcome may be due to the high efficiency of ZnO NRs for light-harvesting based on their optical properties [77,78]. Figure 6c illustrates the variation of the energy gap, absorbance at 490 nm, and photocurrent density at 1 V for all samples. The values of absorbance at 490 nm are 2.03, 1.48, and 1.06 for ZnO NRs, ZnO NDs, ZnO NRs/NDs, respectively. The ZnO NRs film has the lowest energy gap and the highest absorption intensity in the visible light area, and hence possesses the highest PEC current density among the addressed ZnO nanostructured films. Additionally, the nanocrystalline structures in the form of NRs enhance the charge carrier’s separation and thus suppress their recombination [64]. Moreover, ZnO NRs proved efficient charge collection, and good photostability [79,80,81,82]. Besides, the high ZnO NRs surface area allows faster transfer of faradaic charge between semiconductor and electrolyte. Therefore, the ZnO NRs sample deposited at 2 h exhibited higher PEC compared with other samples. ZnO NRs possess the best-optimized properties for PEC. Therefore, Au NPs were sputtering on the ZnO NRs. Figure 6b–d indicates the PEC of the Au/ZnO NRs. In the dark, the O_2_ molecule will adsorb on the ZnO NRs surface and obtain a free electron. This electron will diffuse from the ZnO NRs layer to the Au NPs and a well-formed Schottky barrier layer, which is a typical feature of Au/ZnO [78]. Under illumination, the J_ph_ of Au/ZnO increases sharply with increasing the Au coating time, as seen in Figure 6b. As the Au NPs thickness increased for the Au/ZnO NRs, this matches well with the reduction in the bandgap and the improvement of visible light absorption, resulting in the manufacture of high numbers of electron–hole carriers. At applied 1 V, the J_ph_ of 4 min Au/ZnO NRs is approximately 7.7 mA/cm^2^, which is around 50 times higher than that of pure ZnO NRs (Figure 6c). This means that, for the 4 min Au/ZnO NRs study, which justified the enhanced charge carrier transfer kinetics, less energy was needed to drive the water splitting reaction. Moreover, under zero applied bias (0 V), the Au/ZnO photoanode shows J_ph_ = 0.47 mA/cm^2^, suggesting high water oxidation activity without any support from an external electrical potential source. These results indicate that the coating of Au NPs on ZnO NRs plays a positive role in improving the performance of the photoelectrode, enhances the electrical and optical properties of the ZnO samples, and exhibits enhanced J_ph_ values [83,84]. Au NPs have many significant ways of enhancing PEC performance. The light excites the electrons at the Fermi level of a plasmonic Au NPs and raises it to the localized surface plasmon energy level, as the Fermi level (EF) of Au NPs is lower than the ZnO level. This allows the hot electrons, which resulted from surface plasmon, to be transferred to the ZnO’s conduction band (CB), resulting in increased charge carriers and photocurrent [85,86,87]. The Schottky barrier at the ZnO/Au contact provides, for hot electrons, a unidirectional pathway from Au NPs to the conduction band of ZnO [83]. Consequently, the recombination of the electron–hole is suppressed by this process, leading to better photocatalytic action. Additionally, plasmonic Au NPs were able to broaden the light absorption ability to the visible region [88,89]. The light absorption of Au/ZnO can be broadened into the visible range spectrum due to the plasmonic Au NPs aid, trapping incident light by reducing the scattering and reflection inside the ZnO [86]. In the solar spectrum, the incident photon flux in the visible range is much greater than in the UV region. Hence, the light-capturing in the visible region was a benefit for charge transport and photochemical reactions under simulated sunlight illumination. Additionally, plasmon energy transfer (PET) from plasmon resonant Au surfaces to adjoining ZnO decreases the distance of the travel holes to the electrolyte and, consequently, enhances the J_ph_ value. Additionally, the light-concentrating effect due to the localized strong electric field around the Au NPs can improve the photogeneration rate of electron–hole pairs near the Au/ZnO interface area due to the strengthened electric field intensity [90,91]. Further, the good conductivity of Au NPs over ZnO NRs leads to decreased charge-transfer resistance, suppresses charges recombination, and accelerates the transfer of an electron from the ZnO NRs surface to the electrolyte [53]. Zhang et al. stated that Au/ ZnO core–shell arrays exhibited J_ph_ to be far superior to pristine ZnO photoanode [88]. Therefore, our PEC data refer to the 4 min Au/ZnO NRs sample as an optimum coating for the H_2_ production mechanism. Under the simulated illumination of the Xe lamp, the J_ph_-E curve of the Au/ZnO NRs was observed for six runs, as shown in Figure 6d. The value of the J_ph_ decreased from 7.7 to 6.3 mA/cm^2^ after six runs. This indicates good photochemical stability and reusability of Au/ZnO NRs. Therefore, it can be used as a cost-effective photoelectrode in the practical PEC application.

**Figure 7:**
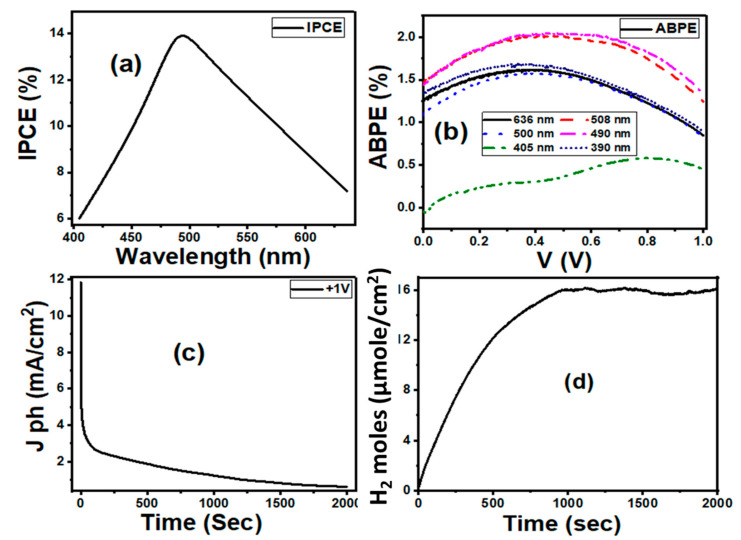
(**a**) IPCE (%) as a function of the wavelength for 4 min Au/ZnO NRs film; (**b**) ABPE (%) as a function of wavelength for 4 min Au/ZnO NRs; (**c**) current–time stability curve under the 400 W Xe Lamp illumination of (**d**) the number of H_2_ moles produced as a function of time.

To examine the PEC efficiency of 4 min Au/ZnO NRs to the incident light absorption, the incident-photon-to-current conversion efficiency (IPCE) was calculated under monochromatic radiance conditions, as demonstrated in Figure 7a. The IPCE value is a calculation of the ratio of the number of photoelectrons used in the redox reactions to the number of incident monochromatic photons on the active site of the electrode [89]. A better value of IPCE indicates the enhanced generation of photoexcited charge carriers. The IPCE value was estimated at an applied external bias of 1 V from Equation (13) [92].

(13) $$\begin{array}{ll} \mathrm{IPCE} & =\frac{\mathrm{total}\;\mathrm{energy}\;\mathrm{of}\;\mathrm{converted}\;\mathrm{electrons}}{\mathrm{total}\;\mathrm{energy}\;\mathrm{of}\;\mathrm{incident}\;\mathrm{photons}}\\ & =\frac{J_{\mathrm{ph}}\left(\mathrm{mA}/{\mathrm{cm}}^{2}\right)}{P_{\mathrm{light}}\left(\mathrm{mA}/{\mathrm{cm}}^{2}\right)}\;\frac{1240}{\lambda \;\left(\mathrm{nm}\right)}\times 100\;\left(\%\right) \end{array}$$

where $J_{\mathrm{ph}}$ (mA/cm^2^) is taken at a certain wavelength for incident light; λ is the wavelength of the radiating monochromatic photon, and $P_{\mathrm{light}}$ is the illuminating light power density. The IPCE value for 4 min Au/ZnO NRs electrode increased with increasing the wavelength of the incident photon until it reached 14.2% at 490 nm and then decreased with increases in the wavelength, as displayed in Figure 7a. In the visible region, the maximum value of IPCE confirms that the Au NPs have tuned the optical absorption.

The applied bias photoconversion efficiency (ABPE) was computed to further examined the performance of the 4 min Au/ZnO NRs using Equation (14) [90].

(14) $$\mathrm{ABPE}=J_{\mathrm{ph}}\left(\mathrm{mA}/{\mathrm{cm}}^{2}\right)\;\frac{V_{\mathrm{redox}}-E_{\mathrm{RHE}}}{P_{\mathrm{light}}\left(\mathrm{mA}/{\mathrm{cm}}^{2}\right)}\times 100\;\left(\%\right)$$

where $V_{\mathrm{redox}}$ is the H_2_O splitting redox potential for (1.23 V) and ${\;P}_{\mathrm{light}}$ is the power of light intensity. ABPE can offer analytical measurements to represent the development of the photoanode performance concerning the externally applied potential. As demonstrated in Figure 7b, the best value of ABPE efficiency attained 2.05% at 490 nm and 1 V. Both IPCE and ABPE have a maximum value at 490 nm. This wavelength is very near to the SPR absorption area for Au NPs [91]. As result, the observed improved PEC performance could be attributed to the plasmonic-enhanced light absorption with a huge increase in the charge generation.

The high photostability of the Au/ZnO NRs over elongated time is another key factor for the enhancement of an efficient PEC hydrogen production system. Figure 7c presented the time-dependent J_ph_ values (J_ph_–t curve). In the first period, the value of J_ph_ is quickly decayed as a result of the minimal photocorrosion process [93]. Above 100 s, the J_ph_ values exhibit a very stable photoresponse at nearly 1.5 mA/cm^2^ due to the rise in the collection of the ionic charges. This indicates that the Au/ZnO NRs have high chemical stability after long-term PEC water splitting. This is because the Au NPs over ZnO NRs can significantly isolate the electron–holes, protect the surface from photocorrosion, and stability can be increased.

The number of hydrogen moles produced can be calculated as follows from the electrolysis law of Faraday:

(15) H2moles=2 ∫Jph dtF

where F is Faraday constant (96500 C/mol), J_ph_ is the photocurrent density in-unit A/cm^2^, and t is the time in sec. The generated number of H_2_ moles as a function of the generation time is shown in Figure 7d, which is calculated from the amperometric J_ph_-t curve using Equation (15). As the generation time increased, the generated number of hydrogen moles per cm^2^ reached an almost steady-state value (16 μmol) after 1000 s. The accumulated number of the generated hydrogen moles per unit area per unit time was 26.622 mmol h^−1^ cm^−2^.

Finally, the PEC performance was obtained in this study compared with many previously reported photoelectrodes as shown in Table 2. The values of J, IPCE, and ABPE confirmed that the 4 min Au/ZnO is effective for PEC water splitting under visible light irradiation. Therefore, it has been concluded that the 4 min Au/ZnO electrode is very suitable for the PEC reactor.

### 3.3. Mechanism of PEC

**Figure 8:**
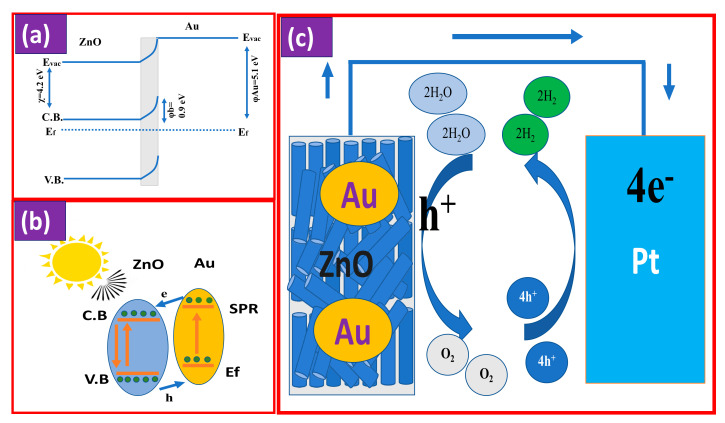
Mechanism of PEC by Au/ZnO photoelectrode;(**a**) band bending, (**b**) energy diagram, and (**c**) PEC water splitting schematics.

The H_2_O splitting reaction on Au/ZnO photoelectrode is presented in Figure 8. At the interface ZnO-Au, the Schottky barrier is formed and the energy band of ZnO is bending upward, Figure 8a. The work function of ZnO is approximately 5.3 eV, which is greater than that of Au (5.1 eV) [106]. This leads to the draft of the electrons from Au to ZnO until the Fermi level is balanced [107]. Under the stimulated light, the electrons of ZnO are excited from the valence band (V.B) to the conduction band (C.B) level with producing the holes in the lower V.B. At the same time, the photoexcitation of Au NPs occurs because the surface plasmonic resonance (SPR) phenomenon, and consequently, the electrons of Au NPs, jump to the Fermi level. These hot electrons can overcome the Schottky barrier and then arrive at the C.B of ZnO NRs, as seen in Figure 8b. This produces holes in the Au NPs and accumulates a greater number of electrons into the C.B of the ZnO conduction band. Moreover, an oscillating electric field of the SPR prevents the recombination of electron–hole pairs and increases the lifetime of charge carriers. Additionally, the electric field that is formed in the space charge region results in band bending. Band bending acts as a barrier to the recombination of charge carriers. Eventually, these electrons transfer through the electric circuit to the Pt foil and accelerate the reduction in H^+^ to generate H_2_ gas, as clear in Figure 8c.

## 4. Conclusions

In this paper, we have synthesized ZnO and Au/ZnO nanostructures with controllable nanomorphologies (NDs, NDs/NRs, and NRs) for water splitting under solar light. The SEM images show that deposition time has a strong effect on the ZnO nanomorphology. ZnO NRs with an average length of 611 nm and a diameter of 210 nm are formed after 2 h, whilst hexagonal nanodiscs with diameters ranging from 380 to 1100 nm are obtained as the deposition time increased from 6 h to 12 h. The XRD findings show that the ZnO has a hexagonal phase with preferential orientation alongside the (002) plane. The Eg value of ZnO NRs (3.19 eV) is reduced with increasing the Au deposition time to 2.07 eV @ 4 min. The 4 min Au/ZnO NRs electrode displayed the best PEC performance: J_ph_ = 7.7 mA/cm^2^ @ 1 V, IPCE =14.2% and ABPE = 2.05% at 490 nm, which is the optimized wavelength. The results show that the 4 min Au/ZnO NR can be applied effectively for the development of cost-effective and large-scale photoelectrodes for devices converting solar energy to hydrogen.

## Figures and Tables

**Table 1:** The XRD parameters for the ZnO films and 4 min Au/ZnO film.

Parameter	ZnO @ 2 h	ZnO @ 6 h	ZnO @ 12 h	4 min Au/ZnO @ 2 h
**Plane (hkl)**	(002)	(002)	(002)	(002)
**2θ**	34.41	34.40	34.39	34.93
**d-spacing (Å)**	5.2976	5.2992	5.3010	2.57
**D (nm)**	79	79	77	65
**A (Å)**	3.2459	3.2357	3.2386	3.2046
**C (Å)**	5.2078	5.2653	5.2488	5.1405
**V (Å^3^)**	47.5176	47.7392	47.6748	45.7176
**L (Å)**	1.9757	1.9785	1.9776	2.2339
**U**	0.37936	0.37576	0.37678	0.3795
**σ** **∗** **10^9^ (N/m^2^)**	−0.2091	−5.17566	–3.74764	5.6131

**Table 2:** Comparison of the values of (J_ph_, IPCE, and ABPE) for the present work with many previous studies values based on ZnO.

Catalyst	J_ph_ (mA/cm^2^)	IPCE %	ABPE %	Ref.
ZnO/Ag	0.616	0.5 @ 390 nm	0.81	[94]
(ZnO/Co)O:N nanowires	8.78 at 0.78 VRHE	15 @ 500 nm	1.39	[95]
C-doped ZnO porous nanoarchitectures	1 at 1 VAg/AgCl	0.75	-	[96]
Au-coated 3D ZnO nanowires	1.45 at 1 VRHE	35	0.52	[48]
ZnO nanorods	0.19 at 1 VSCE	-	0.084	[97]
ZnO-ZnS core–shell	0.534 at 1 VAg/AgCl	-	0.38	[98]
Si/ZnO core–shell	4 at 1 VAg/AgCl	-	0.47	[99]
ZnFe_2_O_4_/ZnO system	1.1 at 0.8 VAg/AgCl	0.15@500 nm	0.15	[100]
Ag/Co-doped ZnO nanowire	0.7 at 0.39 VAg/AgCl	2.6	0.73	[101]
MoS_2_-decorated ZnO nanowires	0.22 at 1 VAg/AgCl	-	3.18	[102]
ZnO/C Core-Shell Nanorods	0.82 at 1 VAg/AgCl	3.5 at 500 nm	0.79	[103]
ZnO:Cu photoanode	2.5 at 1 VSCE	42 at 360 nm	0.28	[104]
Au decorated ZnO:Cu NRs	0.9 at 1 VAg/AgCl	40	0.59	[105]
Au/ZnONRs	7.7	14.2@490 nm	2.05	Present work
